# Experiences of frontline nurses with adverse medical events in a regional referral hospital in northern Ghana: a cross-sectional study

**DOI:** 10.1186/s41182-019-0163-8

**Published:** 2019-05-28

**Authors:** Robert Kaba Alhassan, Bilson Halilu, Saeed Mohammed Benin, Bentor Francis Donyor, Abubakar Yussuf Kuwaru, Dudu Yipaalanaa, Edward Nketiah-Amponsah, Martin Amogre Ayanore, Aaron Asibi Abuosi, Agani Afaya, Solomon Mohammed Salia, Japiong Milipaak

**Affiliations:** 1 Department of Public Health Nursing, School of Nursing and Midwifery, University of Health and Allied Sciences, Ho, Ghana; 2 School of Nursing and Midwifery, University of Health and Allied Sciences, Ho, Ghana; 3 Department of Economics, University of Ghana, Legon, Ghana; 4 School of Public Health, University of Health and Allied Sciences, Hohoe, Ghana; 5 Department of Health Services Management and Public Administration, University of Ghana Business, University of Ghana, Legon, Ghana

**Keywords:** Adverse medical events, Professional nurses, Auxiliary nurses, Regional referral hospital, Northern Ghana

## Abstract

**Background:**

Adverse medical events (AMEs) are threats to delivery of quality healthcare services, particularly in resource-poor settings such as Ghana. In sub-Saharan Africa, 30% of deaths are attributed to AMEs and a significant proportion of these events are not reported. This study explored personal experiences of nurses with AMEs and the constraints to reporting them.

**Methods:**

This is a descriptive cross-sectional study among professional (*n* = 133) and auxiliary (*n* = 88) nurses in a regional referral hospital in northern Ghana. A test for differences in experiences of professional and auxiliary nurses was done using Wilcoxon Mann-Whitney test. Ordered logistic regression analysis (proportional odds ratio models) and probit regression were used to ascertain the determinants of staff’s knowledge on AMEs and the odds of exposure, respectively.

**Results:**

Overall, knowledge and awareness level on AMEs was average (mean = 3.1 out of the five-point Likert scale of 1 = “Very poor” to 5 = “Excellent”). Knowledge levels among professional nurses (mean = 3.2) were relatively higher than those among auxiliary nurses (mean = 3.0), (*p* = 0.006). The predominant type of AME experienced was wrongful documentation (*n* = 144), and the least experienced type was wrong transfusion of blood and/or intravenous fluids (IVF) (*n* = 40). Male staff had higher odds of experiencing medical errors relative to female staff, OR = 2.39 (95% confidence interval (CI), 1.34–4.26). Inadequate logistics was the most perceived cause of AMEs. Knowledge on types of AMEs was significantly associated with gender of the respondents, OR = 1.76 (95% CI, 1.05–2.94); moreover, male staff had higher odds of knowing AME post-exposure action than female staff, OR = 1.75 (95% CI, 1.04–2.93).

**Conclusion:**

Knowledge levels of nursing staff on AMEs were generally low, and even though exposures were high they were not reported. There is the need to integrate AME modules into the pre-service and in-service training curricula for nurses to enhance their knowledge on AMEs; reporting registers for AMEs should be made available in clinical sites and staff incentives given to those who report AMEs. Lastly, protocols on AMEs should form part of the quality assurance value chain for health facilities to promote compliance.

## Background

Adverse medical events (AMEs) are threats to delivery of quality healthcare services, particularly in resource-poor settings such as Ghana. Adverse medical events are experienced by clinicians, including nurses, on a daily basis which include medication errors, adverse drug reaction, needlestick pricks, falls from a height or due to slippery floor, and transfusion reaction. Adverse medical events as used in this context include untoward events that directly or indirectly affect patients and healthcare staff. Studies have shown that even though healthcare workers suffer on a daily basis from these adverse events, the incidents are often not reported due to poor institutional reporting structures and limited knowledge of healthcare staff on these adverse medical events [1].

A review of the medical literature shows that staff experiences with adverse medical events were not given optimal attention by healthcare managers until the 1999 publication of “*To Err is Human*” by the United States (US) Institute of Medicine (IoM) [2]. This publication created the impetus for greater attention on this erstwhile neglected phenomenon in healthcare facilities. Since the release of the IoM report [2], many countries across the globe have voluntarily implemented adverse medical events reporting systems to promote safety of clients and healthcare providers. Nonetheless, as of December 2006, only 27 countries globally passed legislations, regulations, and executive orders for reporting adverse events in healthcare facilities [3].

Adverse medical events in clinical settings occur in developed and developing countries. For instance, medical errors are the third leading cause of death in the United States of America (USA) [4]. Moreover, the World Health Organization (WHO) [5] revealed that 23% of European Union (EU) citizens claim they have been affected by medical errors and 50–70% of these medical errors were highly preventable.

Available statistics in Africa suggest worst trends on adverse medical events. In sub-Saharan Africa, it has been found that 30% of deaths are attributed to AMEs. A study by Ofori and Bates [6] showed that approximately 21% AMEs were recorded in selected teaching hospitals in Ghana because of acute transfusion reaction [6].

AMEs get more challenging when they are not reported by clinical staff as observed by Gagliardi et al. [7] in their study among physicians, nurses, and other clinical staff in Canada and other countries [8, 9]. A study by Alhassan and Poku [1] among 296 nurses and nurse assistants found that barely 44% of interviewed healthcare professionals reported their recent experience of an adverse event in the course of delivering health services.

In light of these low reporting trends among healthcare staff, Nwokike and Eghan [10] intimated that there is the need for a more comprehensive health safety system that goes beyond adverse events data collection to a greater emphasis on enforcement of better reporting systems that include evaluation, minimization, and communication of risks at every level of the healthcare system.

Notwithstanding the high incidence of AMEs in clinical settings, there is paucity of empirical literature on experiences of nurses, particularly in Ghana, resulting in a knowledge gap on this important health safety concern, particularly within the nursing profession. These observations necessitated this study which explored personal experiences of nurses with AMEs and the correlates of reporting trends among these clinical nurses. The study also examined constraints to reporting AMEs. It is expected that the findings of this study will help hospital managers, clinicians, and health policy makers develop robust reporting systems and enforce existing reporting policies on AMEs within the study setting and beyond.

## Methodology

### Study design/setting

This is a descriptive cross-sectional study conducted among different cadres of nursing staff in a major regional referral hospital in the Upper West Region of Ghana.

The Upper West Region (UWR) is one of the administrative regions located in northern Ghana. The region shares boundaries with Burkina Faso to the north, Upper East region to the east, Ivory Coast to the west, and Northern region to the south. UWR covers a geographical area of approximately 18,478 km^2^ and constitutes about 12.7% of the total land area of Ghana; the region is located in the guinea savannah vegetation belt and has a population of 702,110 (341,182 males and 360,928 females) [11].

The study hospital is a major referral hospital for lower level hospitals within UWR, parts of Northern region and neighboring Burkina Faso. The hospital has a total bed capacity of 181 at the time of conducting this study. Services rendered in the facility include general medical services, ante-natal, post-natal, and maternal care. Other service components include specialist care such as ear nose and throat (ENT), dental, laboratory, and physiotherapy.

### Study population

The study population included all cadres of nursing staff on permanent employment. The regional hospital has a total staff population of 682 at the time of conducting this study. Out of this number, 221 were paramedics, 114 casual workers, and 347 nurses of various categories (Upper West Regional: Hospital Administrative Records, 2018; unpublished). Thus, nursing personnel represented approximately 51% of the total workforce in the hospital.

### Sample size and sampling technique

The sampling technique was a census of all professional and auxiliary nurses across all the units of the hospital. Since the nursing staff population was 347, the entire population served as the target sample size (*n* = 347) for the study. However, three (3) extra questionnaires were printed to take care of instances where staff misplaced their questionnaire. This strategy was precautionary because not all questionnaires were retrieved on the day of visit due to busy schedules of some respondents.

### Inclusion/exclusion criteria

The study included nurses of all categories such as professional and auxiliary nurses (i.e., staff licensed by the Nursing and Midwifery Council of Ghana). Only staff on permanent appointment were eligible for inclusion in the study. Also, staff who worked for at least six (6) months on the day of visit were included in this study to obtain data that is reflective of the true experiences of respondents. Staff on post-retirement contract, student nurses, or nurses on rotation/internship were equally excluded.

### Instruments of data collection

A structured questionnaire, comprising of both closed and open-ended questions, was used for the data collection. Since all the target respondents were literates, the questionnaires were largely self-administered and later followed up by the researchers for retrieval. The data collection instrument comprised of four (4) main sections namely: section A (socio-demographic characteristics and work history), section B (experiences and exposure to AMEs), section C (perspectives on causes of AMEs), and section D (reporting of AMEs). Some of the questions on experiences/exposure to AMEs were dichotomized into “Yes” and “No” while others were ranked on a four-point Likert scale as follows: 1 = “strongly disagree,” 2 = “disagree,” 3 = “agree,” and 4 = “strongly agree.” Questions on knowledge levels on AMEs were measured on a five-point Likert scale of 1 = “very poor,” 2 = “poor,” 3 = “average,” 4 = “good,” and 5 = “excellent.” All 22 Likert scale items were tested for scale reliability, and mean Cronbach’s alpha was found to be 0.81, which is acceptable.

### Reliability and validity

The questionnaire was subjected to peer reviews and one pre-testing to promote its validity and reliability. The pre-testing did not lead to changes in the questionnaire, except correction of few typographical mistakes. Moreover, design of the questionnaire was guided by the research objectives and reviewed literature.

### Data analysis

Data was analyzed using the STATA statistical analysis software (version 12.0, StataCorp, College Station, TX, USA). Field data was first captured with Microsoft Excel, cleaned and coded before exporting to STATA for analysis. Chi-square (*χ*^2^) and Fisher’s exact tests were used for the bivariate analysis of categorical data as appropriate while summary statistics on continuous variables were analyzed using independent Student’s *t* test. A test for differences between professional and auxiliary nurses on the Likert scale items was determined using Wilcoxon Mann-Whitney test.

Un-rotated factor analysis was conducted on the various Likert scale items to aggregate the various Likert scale items into similar components. Thus, seven items on staff knowledge on AMEs were factor-analyzed and three were retained, namely “types AMEs,” “action(s) after AME experience,” and “ability to recognize an incidence of AME.” Questions on staff perspectives on the causes of AMEs were also factor-analyzed to arrive at five retained factors out of ten factors. The five retained factors were “poor communication,” “inadequate staff,” “poor management of previous AMEs,” “inadequate skills on AMEs,” and “inadequate motivation.”

Finally, staff perspectives on barriers to reporting AMEs and the corresponding constraints were factor-analyzed and two factors retained out of five. The retained factors were “access to incidence report book” and “lack of clear reporting system.”

### Outcome variables and covariates

Following the factor analysis, ordered logistic regression analysis (proportional odds ratio models) was performed to ascertain determinants of the key outcome variables of interest. The main outcome variables of interest for the ordered logistic regression were the factor-analyzed proxies on knowledge, perceived causes, and constraints to reporting AMEs. Independent variables in the logistic regression were staff age (18–30 years = 1, otherwise = 0), education (first degree = 1, otherwise = 0), gender (male = 1, otherwise = 0), marital status (married = 1, otherwise = 0), religion (Christianity = 1, otherwise = 0), and work experience (5 years or less = 1, otherwise = 0). All independent variables were dichotomized to create uniform reference points (dummies) and enhance ease of interpretation of the findings.

Determinants of personal experience/exposure to AMEs were explored using probit regression. The main outcome variables were five factor-analyzed components on personal experiences with the various AMEs (yes = 1, no = 0). The five factor-analyzed components were needlestick pricks, equipment-related injuries, falls from slippery floor, medication errors, and falls from height. The independent variables in the probit regression are the same as those used for the logistic regression described earlier.

All independent variables were tested for multicollinearity prior to their inclusion in the regression models, and the mean variance inflation factor (VIF) was 1.26. None of the independent variables had a VIF above 10.0 necessary for exclusion from the regression models. Statistical significance was set at 95% for all analysis.

## Results

### Socio-demographic features of respondents

Over 60% of the respondents were aged 18–30 years. There was no statistically significant difference between professional and auxiliary nurses in terms of age; females dominated the study sample constituting 53% while 63% of the respondents said they were married. More professional nurses (37%) said they were married than auxiliary nurses (26%) (*p* = 0.007). Christians constituted 51%, and 62% of the respondents said they worked for 5 years or less (see Table 1).

**Table 1:** Socio-demographic characteristics of respondents (*n* = 221)

Staff characteristics	Professional category			
	Professional (*n* = 133)	Auxiliary (*n* = 88)	Total (*n* = 221)	*p* value
	f (%)	f (%)	f (%)	
Age				
18–30 years	87 (39)	58 (26)	145 (66)	0.170
31–40 years	39 (18)	3014)	69 (31)	
41–50 years	5 (2)	0 (0)	5 (2)	
51–60 years	2 (1)	0 (0)	2 (1)	
Gender				
Male	61 (28)	43 (19)	104 (47)	0.662
Female	72 (33)	45 (20)	117 (53)	
Marital status				
Married	81 (37)	58 (26)	139 (63)	0.567
Not married	52 (24)	30 (14)	82 (37)	
Religion				
Christianity	78 (35)	35 (16)	113 (51)	0.007*
Others	55 (25)	53 (24)	108 (49)	
Work experience				
5 years or less	81 (37)	55 (25)	136 (62)	0.811
Over 5 years	52 (24)	33 (15)	85 (38)	

Source: field data (2018); legend: professional nurses (include registered general nurses, registered community health nurses, registered midwives and other professional post-basic specialist); auxiliary nurses (include nurse assistant clinical, nurse assistant preventive auxiliaries); f (frequency)

*Fisher’s exact

### Knowledge and experiences with adverse medical events

A discretionary cut-off point for higher knowledge was a range of 4.01–5.00; average knowledge ranged from 3.01–4.00, and low knowledge was 2.01–1.00. Summated mean score for knowledge and awareness of AMEs was 3.1 out of the five-point scale. Knowledge levels among professional nurses was significantly higher (mean = 3.2) than those among auxiliary nurses (mean = 3.0), (*p* = 0.006). Respondents expressed better knowledge on “recognition of exposure to adverse medical events” (mean = 3.3, SD = 1.0). It was found that professional nurses demonstrated better (mean = 3.4, SD = 0.96) knowledge in “recognition of exposure to AMEs” than auxiliary nurses (mean = 3.1, SD = 1.10), *p* = 0.019. On the whole, respondents were least informed in the area of “employer’s role in preventing adverse medical events” (mean = 2.8, SD = 1.0) (see Table 2).

**Table 2:** Knowledge on adverse medical events (*n* = 221)

Knowledge proxies on AMEs	Professional category			
	Professional (*n* = 133)	Auxiliary (*n* = 88)	Total	*p* value
	Mean (SD)**	Mean (SD)	Mean (SD)	
Types of AMEs	3.3 (0.97)	3.1 (1.10)	3.2 (1.00)	0.0819
Risk to exposure to AMEs	3.4 (0.92)	3.0 (0.92)	3.2 (0.93)	0.0055*
Advocacy on AMEs	3.2 (0.92)	2.9 (0.97)	3.1 (0.95)	0.0190*
Reporting of AMEs	3.2 (0.99)	3.2 (1.10)	3.2 (1.00)	0.7996
Actions following exposure to AMEs	3.1 (0.97)	2.9 (0.94)	3.0 (0.96)	0.2201
Employer’s role on AMEs	3.0 (1.00)	2.8 (1.10)	2.9 (1.00)	0.0889
Recognition of exposure to AMEs	3.4 (0.96)	3.1 (1.10)	3.3 (1.00)	0.0548
Overall knowledge score on AMEs	3.2 (0.70)	3.0 (0.70)	3.1 (0.71)	0.0346*

Source: field data (2018)

Legend:

*Wilcoxon Mann-Whitney two-tailed test of hypothesis at 95% confidence level

**Means and standard deviations derived from the summated 5-point Likert scale items on knowledge levels on AMEs from 1 “very poor” to 5 “excellent;” thus, higher mean scores depict better knowledge levels on AMEs and vice versa

**Fig. 1:**
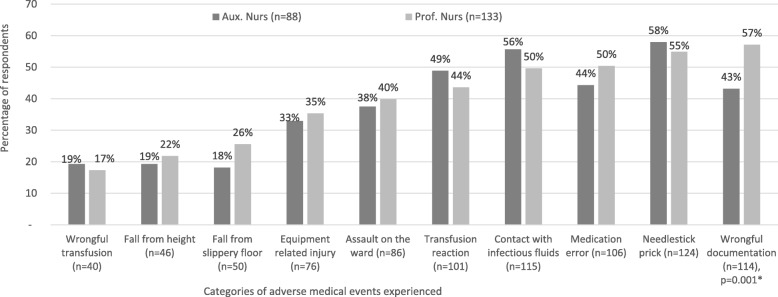
Experiences with adverse medical events in the last 1 month (*n* = 221). Source: field data (2018); legend: Aux. Nurse (auxiliary nurses); Prof. Nurse (Professional nurse); *n* (number of valid responses). Note: percentages are proportion of professional and auxiliary nurses who experienced each of the AMEs. The denominators as the total number of auxiliary (*n* = 88) and professional nurses (*n* = 133) respectively; *Fisher’s exact test statistically significant at 95% confidence level.

Furthermore, it was discovered that the predominant AMEs experienced by the respondents were needlestick prick (*n* = 124), client reaction to transfusion (*n* = 115), wrongful documentation (*n* = 114), and transfusion reaction (*n* = 101). The least AMEs experienced were assault on the ward (*n* = 86), equipment-related injuries (*n* = 76), falls from slippery floors (*n* = 50) or height (*n* = 46), and wrongful transfusion (*n* = 40). In terms of proportions, more professional nurses (57%) experienced wrongful documentation than auxiliary nurses (43%), *p* = 0.001; conversely, needlestick exposures were marginally higher among auxiliary nurses (58%) than professional nurses (55%), albeit the difference is not statistically significant. Likewise, more auxiliaries than professionals reported experience with transfusion reaction by clients, contact with client body fluids, and wrongful administration of transfusion, but the differences were not statistically significant (see Fig. 1).

### Perceived causes of adverse medical events and reporting constraints

**Fig. 2:**
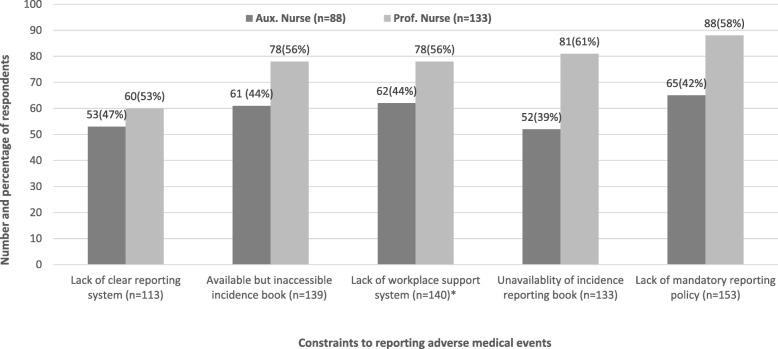
Constraints to reporting adverse medical events (*n* = 221). Source: field data (2018); legend: Aux. Nurse (auxiliary nurses); Prof. Nurse (Professional nurse); *n* (number of valid responses)

Inadequate logistics in clinical settings was scored highest as the prime cause of adverse medical events (mean = 3.3 ± 0.85). The least scored cause of adverse medical events was inadequate requisite skills of working staff (mean = 2.8 ± 0.92). Other identified causes were improper management of previously reported AMEs, low staff motivation, inadequate number of staff, improper documentation, poor communication among staff, poor monitoring and supervision, and improper ward management (see Fig. 2).

**Fig. 3:**
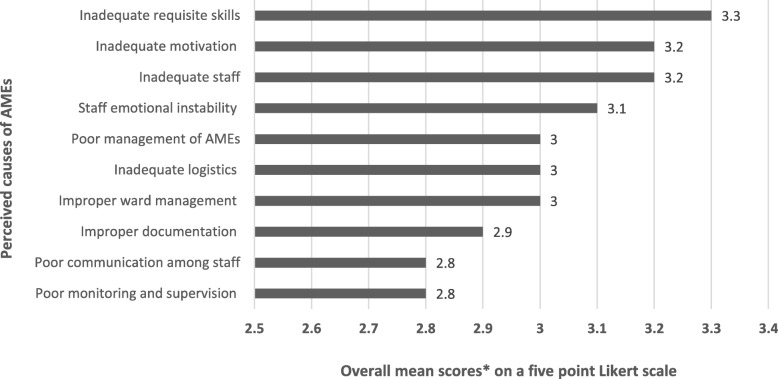
Perceived causes of adverse medical events (*n* = 221). Source: field data (2018); legend: AMEs (adverse medical events); *n* (number of valid responses); SD (standard deviation); *Means and standard deviations derived from the summated five-point Likert scale items on perceived causes of AMEs from 1 “strongly disagree” to 5 “strongly agree;” thus, higher mean scores depict higher magnitude of perceived cause of AMEs and vice versa

Out of the 221 staff interviewed, 153 mentioned lack of mandatory reporting policy in their facility as the constraint to reporting AMEs; 140 staff mentioned lack of workplace support system as a constraint to reporting AMEs, more professional nurses (44%) identified this as a constraint than auxiliary nurses (56%) (*p* < 0.05). Other constraints mentioned were available but inaccessible AME reporting book (139 out of 221), unavailability of AME reporting book (133 out of 221), and lack of clear reporting system for AMEs (113 out of 221) (see Fig. 3).

### Determinants of staff knowledge and exposure to AMEs

It was discovered that knowledge on types of AMEs was significantly associated with gender of the respondents, OR = 1.76 (95% confidence interval (CI), 1.05–2.94); thus, being a male staff increased the odds of staff knowing the types of AMEs relative to females. Likewise, being a male staff improved the odds of knowing the post-AME exposure action than being a female, OR = 1.75 (95% CI, 1.04–2.93). Overall, knowledge on all components of AMEs was correlated with being a male staff, OR = 1.97 (95% CI, 1.19–3.25), and being a Christian by religion, OR = 1.89 (95% CI, 1.17–3.05). Odds ratios were all adjusted for possible effect of confounding variables which were all controlled in the regression analysis. Significant covariates controlled in the regression model were respondents’ age, gender, marital status, religion, and years of work experience. Factors such as professional category, staff age, years of work experience, and marital status did not seem to have a significant association with knowledge levels on AMEs (see Table 3).

**Table 3:** Determinants of staff knowledge levels on adverse medical events (*n* = 221)

Independent variables	Univariate model 1	Univariate model 2	Univariate model 3	Univariate model 4
	Type of AMEs	Post exposure action	AMEs recognition	Overall knowledge
	OR^+^ (95% CI)	OR (95% CI)	OR (95% CI)	OR (95% CI)
Professional category				
Auxiliary nurse	0.72 (0.43–1.19)	0.75 (0.45–1.23)	0.62 (0.37–1.03)	0.65 (0.40–1.05)
Professional nurse	Ref	Ref	Ref	Ref
Age				
18–30 years	0.91 (0.48–1.75)	1.25 (0.66–2.37)	1.09 (0.58–2.06)	1.19 (0.65–2.20)
31 years +	Ref	Ref	Ref	Ref
Gender				
Male	1.76 (1.05–2.94)*	1.75 (1.04–2.93)*	1.46 (0.87–2.44)	1.97 (1.19–3.25)*
Female	Ref	Ref	Ref	Ref
Marital status				
Married	0.64 (0.37–1.10)	1.22 (0.70–2.10)	0.68 (0.39–1.17)	0.82 (0.49–1.38)
Not married	Ref	Ref	Ref	Ref
Religion				
Christian	1.57(.96–2.60)	1.42 (0.86–2.32)	1.13 (0.68–1.88)	1.89 (1.17–3.06)*
Moslem	Ref	Ref	Ref	Ref
Work experience				
5 years or less	0.64 (0.34–1.20)	0.79 (0.42–1.45)	0.59 (0.32–1.10)	0.57 (0.31–1.03)
Over 5 years	Ref	Ref	Ref	Ref
Model statistics				
LR *χ*^2^ (6)	15.34	6.45	8.12	18.64
Prob > *χ*^2^	0.0090	0.2648	0.1500	0.0022
Pseudo *R*^2^	0.0247	0.0107	0.0131	0.0145
Log likelihood	− 302.24	− 298.52	− 305.66	− 635.37

Source: field data (2018)

Legend: OR (odds ratio); *n* (number of valid responses)

*ordered logistic regression significant, *p* < 0.05

+Odds ratios (OR) are all adjusted for possible confounding covariates (i.e., age, gender, marital status, religion, and work experience)

In terms of the determinants of exposure to AMEs, it was found that male staff had higher odds of exposure to medical errors relative to female staff, OR = 2.39 (95% CI, 1.34–4.26). Exposure to falls from slippery floor was found to be correlated with being a Christian relative to other religious faiths, OR = 2.45 (95% CI, 1.22–4.90). Counter intuitively, professional category, gender, age, and years of work experience did not have a significant association with exposures such as needlestick pricks, equipment-related injuries, medication errors, and falls from heights (see Table 4).

**Table 4:** Determinants of exposure to adverse medical events (*n* = 221)

Independent variables	Univariate model 1	Univariate model 2	Univariate model 3	Univariate model 4	Univariate model 5	Univariate model 6
	Need pricks	Equipment injuries	Falls from slippery floors	Medication errors	Falls from height	Overall exposure
	OR^+^ (95% CI)	OR (95% CI)	OR (95% CI)	OR (95% CI)	OR (95% CI)	OR (95% CI)
Professional category						
Auxiliary nurse	1.28 (0.72–2.25)	0.96 (0.53–1.72)	0.75 (0.37–1.50)	0.68 (0.38–1.21)	0.87 (0.44 1.74)	1.03 (0.09 12.52)
Professional nurse	Ref	Ref	Ref	Ref	Ref	Ref
Age						
18–30 years	0.57 (0.28–1.18)	0.89 (0.421.87)	0.67 (0.27–1.65)	0.72 (0.34–1.51)	1.52 (0.63 3.70)	0.61 (0.02 19.23)
31 years+	Ref	Ref	Ref	Ref	Ref	Ref
Gender						
Male	1.00 (0.57–1.77)	1.274 (0.71–2.29)	1.10 (0.56–2.18)	2.39 (1.34–4.26)*	0.79 (0.39 1.58)	0.74 (0.06 9.13)
Female	Ref	Ref	Ref	Ref	Ref	Ref
Marital status						
Married	0.74 (0.40–1.37)	1.04 (0.56–1.96)	0.81 (0.39–1.65)	1.24 (0.67–2.31)	1.04 (0.50 2.18)	1.35 (0.10 18.65)
Not married	Ref	Ref	Ref	Ref	Ref	Ref
Religion						
Christian	1.74 (0.99–3.05)	1.46 (0.82–2.61)	2.45 (1.22–4.90)*	0.68 (0.39–1.20)	1.08 (0.55 2.13)	omitted**
Moslem	Ref	Ref	Ref	Ref	Ref	Ref
Work experience						
5 years or less	1.66 (0.83–3.35)	9.91 (0.45–1.87)	1.93 (0.80–4.68)	1.86 (0.91–3.82)	0.88 (0.38 2.02)	1.80 (0.06 54.08)
Over 5 years	Ref	Ref	Ref	Ref	Ref	Ref
Model statistics						
LR *χ*^2^(6)	7.78	2.54	11.36	17.04	1.99	0.24
Prob > *χ*^2^	0.2546	0.8639	0.0778	0.0091	0.9204	0.9987
Pseudo *R*^2^	0.0257	0.0089	0.0481	0.0557	0.0088	0.0086
Log likelihood	− 147.64	−140.96	− 112.49	− 144.48	− 112.04	− 13.73

Source: field data (2018)

Legend: OR (odds ratio)

*logistic regression statistically significant, *p* < 0.05

**omitted variable from regression model due to collinearity

+Odds ratios (OR) are all adjusted for possible confounding covariates (i.e., age, gender, marital status, religion, and work experience)

## Discussion

This study explored personal experiences of nurses in a major regional referral facility in the UWR of Ghana. In terms of staff self-rated knowledge levels on AMEs, it was found that the average rating was neither good nor excellent on any of the knowledge proxies. The least rated knowledge was on the role of employers in the control and prevention of AMEs. This observation corroborates previous studies on nurses’ experiences with AMEs [1, 6, 7]. Alhassan and Poku [1] and Gagliardi et al. [7] particularly found insufficient knowledge levels among nurses and physicians, respectively, on AMEs. Gagliardi et al. [7] further found significant differences between professional and auxiliary nurses on AMEs. Alhassan and Poku [1] observed that among the nursing staff, professional nurses demonstrated better knowledge on AMEs than their auxiliary colleagues. Perhaps the shorter and less detailed pre-service training offered to auxiliary nurses [12] might account for these differences.

A systematic review involving 53 studies revealed that prevalence estimates for AMEs ranged from a low of 2% to a high of 94% [13]. It was found from this review that inappropriate prescription was the predominant type of error reported. It was also found that the incidence of preventable adverse drug events (ADEs) for instance was estimated to be 15/1000 person-years [13]. A similar review by Mekonenn et al. [14] confirmed the high prevalence of AMEs across the globe. However, many of these previous studies did not emphasize the experiences of healthcare professionals, particularly nurses who are either direct victims or contributing factors to patients’ exposure to these AMEs [15].

In the African context, the numbers are even more overwhelming albeit there is limited empirical literature specifically reported on nurses [1]. In the reviewed literature, cases of AMEs are grossly underestimated since they are often unreported in deprived healthcare facilities due to a litany of reasons [1, 13, 14] including inadequate knowledge on AMEs.

Respondents were also asked about their personal experiences with the different categories of AMEs. It was revealed that the predominant AME was needlestick pricks (*n* = 124) followed by wrongful documentation in the course of rendering care to clients (*n* = 114). The least AME exposure mentioned by the staff was wrong transfusion of blood and/or IVF. Other AMEs mentioned by the respondents were medication error, contact with infectious bodily fluids, transfusion reaction, assault on the ward, equipment-related injuries, and falls. This observation corroborates findings in previous publications where similar AMEs were reported in clinical settings in Ghana [1, 16–18] and other countries [13, 15, 19, 20].

It was also found that auxiliary nurses suffered more of needlestick pricks, contact with infectious bodily fluids, and wrong transfusion. Professional nurses on the other hand experienced more of wrongful documentation, medication error, assault on the ward, equipment-related injuries, and falls. Even though there is no much relevant empirical literature to compare with these current findings, Alhassan and Poku [1] observed similar dynamics in the exposure of professional and auxiliary nurses in two psychiatric hospitals in Ghana.

These observations could be attributed to the differences in training and work mandate of these different cadres of nurses in Ghana. For instance, the relatively higher exposure of professional nurses to documentation, medication errors, and equipment-related injuries could be because these nursing duties are predominantly within the mandate of professional nurses and this could increase the odds of their exposure because they often perform these duties.

Conversely, the higher exposure of auxiliary nurses to transfusion reaction and administration of wrong transfusions could be attributed to instances when these duties are performed on behalf of professional nurses in the form of task shifting due to limited number of qualified professional nurses in Ghana, like many countries in Africa [12, 21, 22]. This phenomenon perhaps reflects the spillover effects of acute shortage of professional nurses in predominantly rural regions in northern Ghana which continue to record lower staff to patient ratios below the national averages [22]. The differences in self-reported exposures by the professional and auxiliary nurses might be attributed to differences in the honesty of staff in reporting these AMEs especially when evidence of professional negligence is implied as argued by Edwin [18] in his study on non-disclosure of medical errors in Ghana.

On the perceived causes of AMEs, inadequate logistics was rated highest while the least perceived cause was staff emotional instability. Other perceived causes were inadequate staff numbers, poor monitoring and supervision, inadequate skills of staff, improper ward management, poor staff motivation, unsatisfactory management of previous AMEs, poor communication among staff, and improper documentation. These responses support similar conclusions on causes of AMEs in Ghana [1, 16, 17, 23] and other countries [13, 15, 19, 20]. The responses re-echo the longstanding challenges of most developing health systems in Africa, ranging from human and material resource constraints. The effects of these health system challenges on the safety of staff and clients have been confirmed in this study and similar studies in the past [1, 5, 13, 15, 24].

In terms of the constraints to reporting exposures to AMEs, majority of the respondents mentioned the absence of a mandatory reporting policy for AMEs. Lack of workplace support system and inaccessible AME reporting books/registers were also identified as major constraints to reporting AMEs. Alhassan and Poku [1] observed in their study among 296 nurses and nurse-assistants in Ghana that majority of nurses did not report AMEs because of unclear reporting system, poor management of previous exposures leading to loss of trust and confidence in facility heads. Similar conclusions were made in previous studies that investigated barriers to reporting AMEs in healthcare facilities [5, 13, 15, 16, 18, 23].

Finally, after controlling for relevant covariates, it was found that gender significantly predicted higher odds of staff knowledge on AMEs and exposure to medication errors. Mrayyan et al. [25] made similar observation in their study on predictors of reporting medication errors in Jordan where gender emerged as a significant correlate. However, Alhassan and Poku [1] in their study on workplace safety among nursing staff in psychiatric hospitals in Ghana did not find statistically significant association between gender and exposure to AMEs. Perhaps the gender effect in this current study could be explained by the fact this study was not conducted in psychiatric facilities as the case in Alhassan and Poku [1]. Moreover, like many countries, the nursing profession in Ghana is female dominated, and since a greater proportion of the professional nurses were females who had lower tendency of committing medication error, the study is not counterfactual in demonstrating that more males had higher odds of experiencing medication errors relative to their female colleagues.

## Conclusion

Overall, this study found that even though nurses experienced AMEs on a daily basis, the victims of these AMEs did not have adequate knowledge in terms of the types, reporting, steps to be taken after exposure, and the role of their employer in AMEs control and prevention. The study also revealed that professional and auxiliary nurses do not experience equal proportions of the different types of AMEs. Thus, some AMEs were more common with professional nurses than with auxiliary nurses. Perceived causes of AMEs were however almost uniformly enumerated by the staff which include inadequate material and human resources, poor monitoring and supervision, and improper ward management and documentation. Staff generally identified lack of mandatory reporting policy for AMEs, inaccessibility of AMEs reporting registers, lack of workplace support, and unclear reporting system as key constraints to reporting AMEs.

### Implications for nursing policy and clinical practice

In light of the above findings, the following policy recommendations are proposed to promote safety of nurses and clients:

1. Pre-service and in-service training curricula for nurses should incorporate detailed modules on AMEs to adequately prepare them ahead of clinical practice. In line with this, protocols for reporting AMEs and registers for reporting AMEs should also be made available and clearly communicated to staff to ensure compliance
2. Incentive packages should also be introduced to reward staff who routinely report incidence of AMEs to promote compliance
3. As part of accreditation processes for healthcare facilities, protocols for monitoring and preventing AMEs should form part of the mandatory requirements for passing the accreditation to encourage compliance by health facilities

### Limitations of the study

This study is based on perceptions of nurses on AMEs. Findings may therefore be different from the reality in practice. In view of this, future studies could review patient medical records retrospectively to ascertain veracity or otherwise of findings of this study.

## Data Availability

All data supporting our findings are contained in the manuscript. There are no restrictions to data and datasets are available from the corresponding author upon reasonable request.

## Abbreviations

ADEs: Adverse drug events
AMEs: Adverse medical events
EU: European Union
IoM: Institute of Medicine
IVF: Intravenous fluid
USA: United States of America
UWR: Upper West Region
VIF: Variance inflation factor
WHO: World Health Organization
